# Clinically significant findings in a decade‐long retrospective study of prenatal chromosomal microarray testing

**DOI:** 10.1002/mgg3.2349

**Published:** 2024-01-23

**Authors:** Joie O. Olayiwola, Mohammad Marhabaie, Daniel Koboldt, Theodora Matthews, Amy Siemon, Danielle Mouhlas, Taylor Porter, George Kyle, Cortlandt Myers, Hui Mei, Ying‐Chen Claire Hou, Melanie Babcock, Jesse Hunter, Kathleen M. Schieffer, Yassmine Akkari, Shalini Reshmi, Catherine Cottrell, Mariam T. Mathew, Marco L. Leung

**Affiliations:** ^1^ The Steve and Cindy Rasmussen Institute for Genomic Medicine Nationwide Children's Hospital Columbus Ohio USA; ^2^ Department of Pathology The Ohio State University of College of Medicine Columbus Ohio USA; ^3^ Department of Pediatrics The Ohio State University of College of Medicine Columbus Ohio USA

**Keywords:** amniocentesis, aneuploidy, chromosomal microarray, genomics, prenatal testing, products of conception

## Abstract

**Background:**

Chromosomal microarray (CMA) is commonly utilized in the obstetrics setting. CMA is recommended when one or more fetal structural abnormalities is identified. CMA is also commonly used to determine genetic etiologies for miscarriages, fetal demise, and confirming positive prenatal cell‐free DNA screening results.

**Methods:**

In this study, we retrospectively examined 523 prenatal and 319 products‐of‐conception (POC) CMA cases tested at Nationwide Children's Hospital from 2011 to 2020. We reviewed the referral indications, the diagnostic yield, and the reported copy number variants (CNV) findings. Results.

In our cohort, the diagnostic yield of clinically significant CNV findings for prenatal testing was 7.8% (*n* = 41/523) compared to POC testing (16.3%, *n* = 52/319). Abnormal ultrasound findings were the most common indication present in 81% of prenatal samples. Intrauterine fetal demise was the common indication identified in POC samples. The most common pathogenic finding observed in all samples was isolated trisomy 21, detected in seven samples.

**Conclusion:**

Our CMA study supports the clinical utility of prenatal CMA for clinical management and identifying genetic etiology in POC arrays. In addition, it provides insight to the spectrum of prenatal and POC CMA results as detected in an academic hospital clinical laboratory setting that serves as a reference laboratory.

## 1 INTRODUCTION

Chromosomal microarray (CMA) is a diagnostic tool that has been integrated into many medical specialties (Mathew et al., 2022). In the pediatric setting, CMA is a first‐tier diagnostic test for individuals with intellectual disability, developmental delay, and multiple congenital anomalies (Miller et al., 2010). In the practice of obstetrics, CMA is primarily performed on samples obtained from amniocentesis, percutaneous umbilical blood sampling, and chorionic villi sampling samples, with or without chromosome analysis (Lo et al., 2014). The diagnostic yield of CMA has been previously demonstrated to be higher than karyotyping in routine prenatal testing because it precludes the need for culturing (Pauta et al., 2018; Raca et al., 2009; Wapner et al., 2012). In addition, the high sensitivity of CMA can detect chromosomal aneuploidies, as well as submicroscopic deletions and duplications, that may be causal for miscarriages, spontaneous abortions, and abnormal fetal ultrasound findings (Donnelly et al., 2014; Edwards & Hui, 2018; Levy & Wapner, 2018; South et al., 2013; Wapner et al., 2012; Zhu et al., 2018).

In addition to abnormal fetal ultrasound findings, CMA can be utilized as a confirmatory test for positive prenatal screening results. Historically, maternal serum screening with or without ultrasound can achieve a detection rate of 80%–95% for Trisomy 21 (Rose et al., 2020). Prenatal cell‐free DNA screening was later introduced in 2011; it uses cell‐free DNA to detect common fetal aneuploidies with high sensitivity and specificity (Rose et al., 2022). Recently, ACMG recommended prenatal cell‐free DNA screening for fetal trisomies 13, 18, 21 as well as fetal sex chromosome aneuploidy, over traditional screening methods for all pregnant individuals (Dungan et al., 2023). Confirmation testing using a diagnostic procedure, such as chromosomal analysis or CMA is recommended for positive screening test results confirmation due to risk for false positives and varying positive predictive values with increasing maternal age (Cherry et al., 2017; Liehr, 2022).

Moreover, genetic testing using CMA on products of conception (POC) is also common in obstetric practice. It can aid in clinical management by identifying a genetic etiology or refining recurrence risk for families. Previous studies have demonstrated that CMA could identify chromosome anomalies in approximately 60% of POC cases from individuals with a single or recurrent pregnancy loss (Dahdouh & Kutteh, 2021; Smits et al., 2020). However, given that POC testing recommendations vary by professional organizations, a broad study of POCs with varying reasons is uncommon (Papas & Kutteh, 2021; Schilit et al., 2022).

Our laboratory serves as a reference laboratory for CMA testing for surrounding hospitals in central Ohio. In this retrospective study, we provide our diagnostic yield and the copy number variant (CNV) findings of prenatal and POC CMA for the past decade. We describe the clinical testing indications and discuss the frequent findings in our study cohort.

## 2 MATERIALS AND METHODS

### 2.1 Data collection and analyses

The data was generated from a single study site, the Institute for Genomic Medicine at Nationwide Children's Hospital (NCH). Retrospective data analysis of clinical arrays consisting of 523 prenatal (517 cultured amniocytes, 3 direct amniotic fluid, 2 fetal/cord blood, 1 pleural effusion) and 319 POC microarray cases extracted from NCH's laboratory reporting software (CoPathPlus, Sunquest Information Systems, Tucson, AZ) from January 1st, 2011, to December 31, 2020, via a data exploration tool. Prenatal arrays were clinically offered beginning in August 2011, while POC arrays were offered beginning in September 2012. Canceled tests (due to poor DNA quality, insufficient fetal tissue, maternal fetal contamination, etc), and proficiency testing samples were excluded from this study. The clinical data generated included the size and chromosome bands of the losses and gains, referral indications, and the clinical significance of each finding.

CMA analysis was performed on two microarray platforms over that ten‐year period: Signature NimbleGen 135K oligonucleotide array for 14 POC arrays from September 2012 to December 2014, and 55 prenatal arrays from August 2011 to March 2014 [Signature Genomics, Spokane, WA] and Agilent 180k CGH+SNP array for 305 POC arrays from January 2015 to December 2020, and 468 prenatal arrays from April 2014 to December 2020 [Agilent Technologies, Santa Clara, CA]. DNA was extracted from cultured and uncultured amniocytes, cultured tissue, fresh tissue, and snap‐frozen tissue. CNV analyses were performed using Genoglyphix software (PerkinElmer, Waltham, MA). CNV calls were made when five consecutive probes were present in the coding region of a gene.

We also utilized the UCSC Genome Browser LiftOver tool to convert hg18 genomic coordinates to hg19 for older cases (http://www.genome.ucsc.edu/cgi‐bin/hgLiftOver) (Kent et al., 2002; Raney et al., 2013). The genome coordinates listed are Human Genome Reference Consortium Build GRCh37 build. All graphs were generated with GraphPad Prism software v.9.0.0 (GraphPad Software, San Diego, CA), and tables were generated with Microsoft Word and Excel (Microsoft Corporation, Redmond, VA). The karyotype figure was adapted from “Human Karyotype” created with BioRender.com (Toronto, Ontario, 2023).

## 3 RESULTS

**FIGURE 1:**
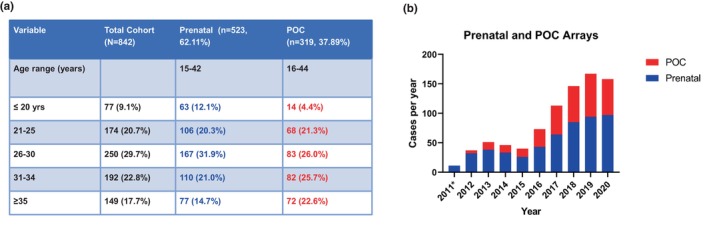
Test demographics and case intake. (a) Patient Demographics by age. A total of 842 patients who underwent prenatal or products of conception (POC) microarray tests in our cohort are categorized into five different age groups. The ages ranged from 15–44 years for both prenatal (blue) and POC arrays (red). (b) Distribution of prenatal and POC arrays received annually at this institution. The POC cases are in red, while the prenatal cases are in blue. *In 2011, there were no POC arrays ordered; the legend indicates prenatal arrays are represented in blue and POC arrays in red.

Our cohort consisted of 842 microarray cases (523 prenatal cases and 319 POC cases) from pregnant individuals ages 15 to 44 years. The majority (29.7%) of the pregnant individuals were ages 26 to 30, while only 77 individuals (9.1%) in our cohort were 20 years of age or younger, and 17.7% of our cohort were ages 35 years or older (Figure 1a). From 2011 to 2015, receipt of prenatal specimens for CMA ranged from 11 to 38 annually, while POC arrays ranged from 0 to 14 cases annually. Beginning in 2016, we observed an upward trend in the order rate for both the prenatal and POC array cohorts. By 2020, we had logged 57 prenatal and 39 POC cases annually (Figure 1b). The average turnaround times for our prenatal and POC testing are 11 days and 14 days, respectively.

**FIGURE 2:**
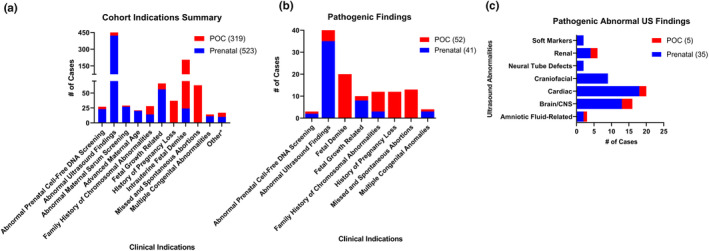
Clinical indications summary and distribution of pathogenic findings. (a) Clinical indications listed on the sample requisition were used to group the samples received. *Other is a category for all indications with no commonalities or lacking a clinical indication. (b) Samples with pathogenic findings (93/842) identified were categorized by clinical indication. The number of pathogenic cases for each array type is the number indicated in the brackets in the legend (c) Pathogenic samples with abnormal ultrasound findings were categorized by organ systems in the five POC and 31 prenatal samples. Multiple cases had more than one clinical indication and/or had multiple organ systems affected, therefore the total number of cases in the distribution may be greater than the total pathogenic cases in this Figure.

To better understand the indication for testing, we stratified our study cohort based on referral indications as indicated on specimen requisitions. The top two indications were abnormal ultrasound findings (*n* = 465/842) and intrauterine fetal demise (*n* = 206/842). Other indications include abnormal prenatal cell‐free DNA screening (*n* = 27), family history of chromosomal abnormalities (*n* = 28), fetal growth abnormalities (*n* = 66), history of pregnancy losses (*n* = 37), maternal serum screening (*n* = 29), missed and spontaneous abortions (63), and multiple congenital anomalies (*n* = 14) (Figure 2a).

The diagnostic yield of this cohort was 11.05% (93/842) similar to other studies (Luo et al., 2021). The prenatal arrays had a diagnostic yield of 7.8% (41/523) while the POC arrays had a diagnostic yield of 16.3% (52/319). When stratified by microarray platforms, the NimbleGen 135K array had 8.7% diagnostic yield (6/69), compared to 11.25% in Agilent 180k array (87/773). There were 544 normal cases, 205 variants of unknown significance findings (VUS) cases, and 93 cases with clinically significant CNV findings (pathogenic). In brief, 136 and 69 VUSes were identified in prenatal and POC samples, respectively. Pathogenic and VUS copy number findings are illustrated in Figure S1 and are listed in Table S1.

Upon examination of the 93 samples with clinically significant findings, the abnormal ultrasound findings category had the highest frequency of cases at 43% (*n* = 40/93) (Figure 2b). Other top indications include intrauterine fetal demise and stillbirths (21.5%, *n* = 20/93), missed and spontaneous abortions (14%, *n* = 13/93), family history of chromosomal abnormalities (12.9%, *n* = 12/93), and recurrent pregnancy losses (12.9%, *n* = 12/93) (Figure 2b). Due to the high rate of clinically significant cases with abnormal ultrasound findings, we further delineated these cases by organ systems (Figure 2c). There were five POCs and 35 prenatal cases with a pathogenic finding with a referral indication of abnormal ultrasound findings. For prenatal arrays, 18 cases had cardiac abnormalities, and 13 cases had brain and central nervous system abnormalities. The remainder were due to various organ system abnormalities including amniotic fluid volume, central nervous system, craniofacial, renal, and soft markers. For POC arrays, three out of the five cases had a central nervous system (CNS) ultrasound abnormality (Figure 2c).

Since cardiac abnormalities were the most common abnormal ultrasound finding in our cohort (18 prenatal and three POC cases), we further detail the clinically significant findings here. The cardiac abnormalities include atrioventricular canal defect, atrioventricular septal defect, coarctation of the aorta, hypoplastic left heart, ventricular septal defect, and tetralogy of Fallot. The CNV findings observed in these cases include 22q11.2 loss (*n* = 3), 6q27 loss (*n* = 2), 1p36 microdeletion (*n* = 2), 15q11.2 loss (n = 1), 16p11.2 loss (*n* = 1), Trisomy 21(*n* = 1), 4q32.2‐q34.2 loss (*n* = 1), 5q34‐35.3 gain (*n* = 1), 10q11.22‐q11.23 gain (*n* = 1), 10q24.2‐q25.1 (*n* = 1), and six cases involving both terminal losses and gains, suggesting unbalanced translocations. Of the 128 cardiac‐related ultrasound findings seen in this cohort, ventricular septal defect (VSD) was the most frequent in this study (20/128), and 20% (4/20) of those VSDs had clinically significant findings, although this was not the only ultrasound finding in all four cases (See Table S2 for the 20 VSD cases and their clinical findings and indications).

**FIGURE 3:**
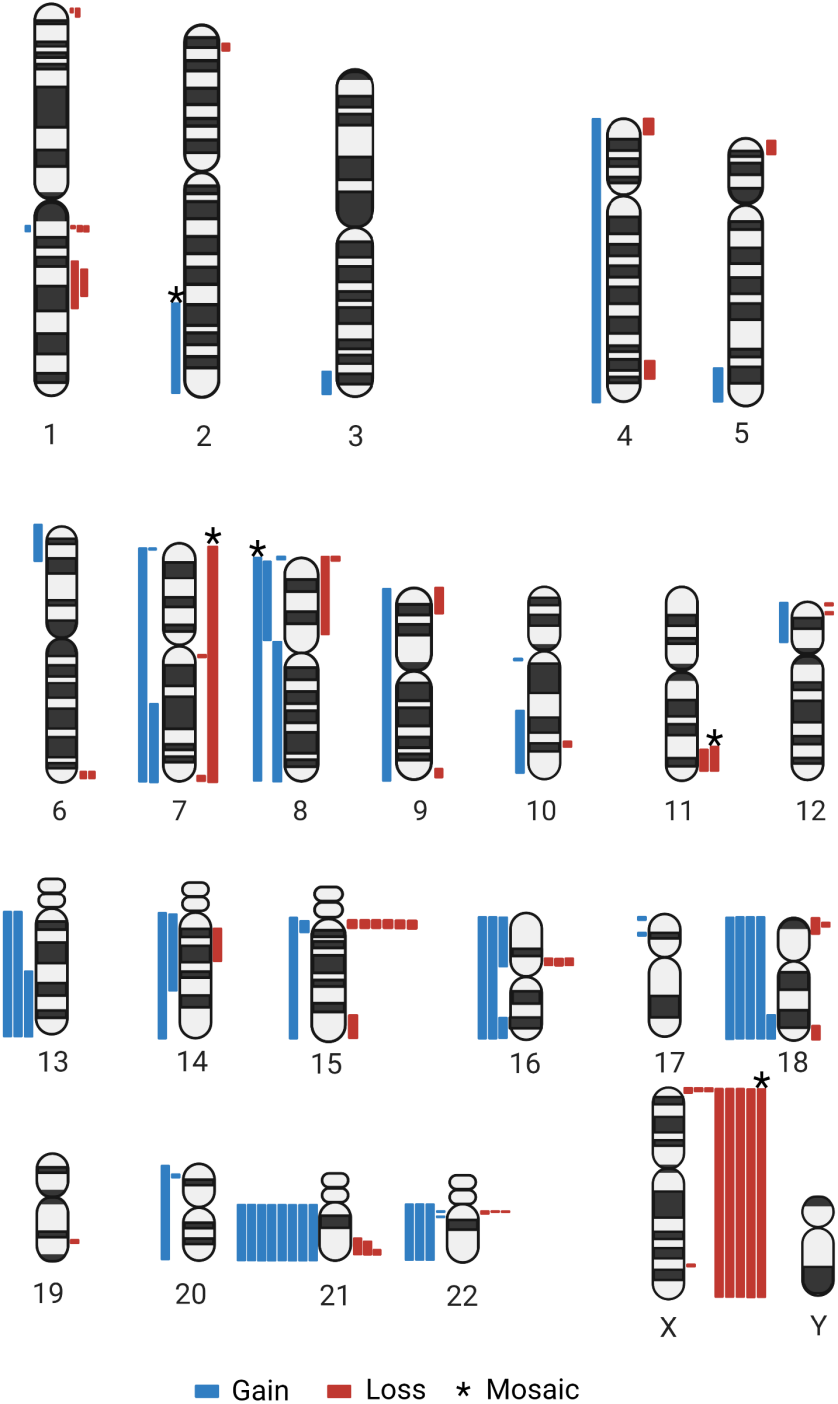
Clinically significant (pathogenic) copy number variants. This ideogram depicts clinically significant CNV findings identified in 93 samples with clinically significant findings reported as likely pathogenic or pathogenic. Please note, the ring chromosome 7 and triploidy cases are not depicted in this ideogram. CNV findings are represented by blue segments (gains) and red segments (losses), mosaic CNV findings are represented with an asterisk above the segment. Multiple cases had two pathogenic CNV findings as noted in Table 1.

The pathogenic findings identified in our cohort are illustrated in Figure 3. Trisomy 21 was most frequently observed aneuploidy (*n* = 7), followed by monosomy X (*n* = 4), trisomy 18 (*n* = 4), and trisomy 13 (*n* = 2). Notably, 66.5% (*n* = 33/52) of the POC samples were aneuploid or polyploid, with 14 samples involving rare autosomal trisomies or triploidy that are not compatible with life. They include triploidy (*n* = 4), trisomy 22 (*n* = 3), trisomy 16 (*n* = 2), trisomy 7 (*n* = 1), trisomy 9 (*n* = 1), trisomy 4 (*n* = 1), trisomies of both 14 and 20 (*n* = 1), and trisomies of both 15 and 21 (*n* = 1). Mosaic findings were also detected in our cohort, including a mosaic trisomy 8, 45,X/46,XX mosaicism, and a case with two mosaic cell lines involving a monosomy 7 and a ring chromosome 7 (Case 20). In addition, both prenatal and POC microarrays also identified common deletion and duplication syndromes including 1p36 deletion syndrome (*n* = 2), Wolf–Hirschhorn syndrome (*n* = 1), Williams‐Beuren syndrome (*n* = 1), Cri‐du‐chat syndrome (*n* = 1), 15q11.2 deletion syndrome (*n* = 6), 16p11.2 deletion syndrome (*n* = 3), 22q11.2 deletion syndrome (*n* = 2), distal 22q11.2 deletion syndrome (*n* = 1), 22q11.2 microduplication syndrome (*n* = 2) (Figure 3 and Table 1).

**TABLE 1:** List of pathogenic CNV findings.

Case #	Pathogenic CNV findings (hg19 coordinates)	CNV size	Clinical indications	OMIM syndrome	Additional cytogenetic findings
1	arr[GRCh37] 1p36.33p36.32(945927_2460720)x1	1.51 Mb	Tetralogy of Fallot, abnormal radius and ulna, hypotelorism, intrauterine growth restriction	1p36 deletion syndrome	
2	arr[GRCh37] 1p36.33p36.22(835650_11715597)x1	10.81 Mb	Microcephaly, abnormal cerebellum, abnormal head shape, hypoplastic left heart	1p36 deletion syndrome	
3	arr[GRCh37] 1q21.1(145400040_145746492)x1	346.45 Kb	Recurrent pregnancy loss & advanced maternal age	Thrombocytopenia‐absent radius (TAR) syndrome	
4	arr[GRCh37] 1q21.1q21.2(146531538_147726542)x3	1.20 Mb	Multiple congenital anomalies	Distal 1q21.1 microduplication syndrome	
5&6	arr[GRCh37] 1q21.1q21.2(146531538_147390104)x1 [2]	858.57 Kb	(1) Asymmetric intrauterine growth retardation (2) Intrauterine growth retardation, poor weight gain	Distal 1q21.1 microdeletion syndrome	
7	arr[GRCh37] 1q24.2q31.3(167337463_197169306)x1	29.83 Mb	Maternal family history of abnormal chromosome 1		
8	arr[GRCh37] 1q25.1q31.2(173172446_191983735)x1	18.81 Mb	Intrauterine growth restriction and cleft lip		
9	arr[GRCh37] 2p24.3p23.3(16150141_24532813)x1	8.38 Mb	35‐week stillborn male fetus with renal agenesis		
10^a^	arr[GRCh37] 2q31.3q37.3(181541196_242980297)x3 mos,11q23.2q25(113658085_131326822)x1 mos	61.44 Mb 17.67 Mb	Pregnancy loss		
11	arr[GRCh37] 3q27.3q29(187614759_197771082)x3,15q26.1q26.3(94231714_102391142)x1	10.16 Mb 8.16 Mb	Congenital diaphragmatic hernia, coarctation of the aorta, thick nuchal fold, and baby measuring small		46,XX normal
12	arr(4)x3	190.82 Mb	Recurrent pregnancy loss	Trisomy 4	
13	arr[GRCh37] 4p16.3p16.1(73000_10876883)x1	10.80 Mb	Club feet, diaphragmatic hernia	Wolf–Hirschhorn Syndrome	
14	arr[GRCh37] 4q32.2q34.2(162562177_177158314)x1	14.61 Mb	Severe intrauterine growth retardation, possible AV canal defect and cardiomegaly		
15	arr[GRCh37] 5p15.33p15.2(55550_11521421)x1,8p23.3p23.2(202133_5249394)x3	11.47 Mb 5.05 Mb	Intrauterine fetal demise	Cri du chat Syndrome	Unconfirmed/possible unbalanced translocation
16	arr[GRCh37] 5q34q35.3(160003237_180686443)x3	20.68 Mb	Fetus with craniosynostosis, pelvic kidney, short femur, two‐vessel cord, and atrioventricular septal defect		
17	arr[GRCh37] Xp22.33p22.31(2699521_7364049)x1,6p25.3p22.3(195378_21614483)x3	7.03 Mb 21.42 Mb	Intrauterine fetal demise		Unconfirmed/possible unbalanced translocation
18	arr[GRCh37] 6q27(164506324_170901286)x1	6.39 Mb	Complex fetal cardiac anomaly		
19	arr[GRCh37] 6q27(164506324_170901287)x1	6.39 Mb	Congenital heart defect, ventriculomegaly, agenesis of corpus callosum, and hypoplastic cerebellum		
20^a^	arr[GRCh37] (7)x1 mos,7q36.3(155104047_159123166)x1 mos	159.08 Mb 4.02 Mb	Closed sacral neural tube defect, dilated 4th ventricle, clubbed feet, and absent right kidney		mos 45,XY,‐7[5]/46,XY,r(7)(p?22q?36)[3]/46,XY[5]
21	arr(7)x3	159.04 Mb	Miscarriage		Karyotype not possible due to culture failure
22	arr[GRCh37] 7p22.3p22.1(176612_5819144)x3	5.50 Mb	Abnormal right kidney and bilateral choroid plexus cysts		
23	arr[GRCh37] 7q11.23(72745047_74138460)x1	1.39 Mb	Intrauterine fetal demise	Williams‐Beuren Syndrome	
24	arr[GRCh37] 7q31.1q36.6(107465993_159123166)x3,21q22.3(46656444_48091215)x1	51.66 Mb 1.43 Mb	Ventriculomegaly, possible ventricular septal defect, and absent cavum septum		Unconfirmed/possible unbalanced translocation
25	arr[GRCh37] 7q36.3(155292541_159123333)x1	3.83 Mb	Holoprosencephaly and micropenis		
26^a^	arr(8)x3 mos	145.80 Mb	Bilateral cleft lip and absent cavum septi pellucidi	Mosaic Trisomy 8^a^	
27	arr[GRCh37] 8p23.3p23.1(202262_6920312)x1,8p23.1p11.21(12583124_43048375)x3	6.72 Mb 30.54 Mb	Prenatal ultrasound abnormalities: midline heart with pericardial effusion, two‐vessel cord, multiple fetal brain abnormalities (agenesis of corpus callosum, interhemispheric cyst, mild ventriculomegaly, posterior fossa communication), fetal MRI showed underdeveloped sulci, and small cerebellum		46,XX,add(8)(p23.1)
28	arr[GRCh37] 8p23.3p11.23(202133_38071089)x1,8p11.21q24.3(39749478_146293435)x3	37.87 Mb 106.54 Mb	Recurrent spontaneous first trimester pregnancy losses		Karyotype not possible due to culture failure
29	arr(9)x3	140.77 Mb	Recurrent pregnancy loss	Trisomy 9	
30	arr[GRCh37] 9p24.3p23(209020_9841683)x1	9.63 Mb	Intrauterine growth restriction with choroid plexus		
31	arr[GRCh37] 9q34.3(137726419_141005513)x1,16q23.3q24.3(81907540_90119719)x3	3.28 Mb 8.21 Mb	Anencephaly, rocker bottom feet, and clenched fists		46,XY,der(9)t(9;16)(q34;q23)
32	arr[GRCh37] 10q11.22q11.23(46980161_51595050)x3	4.61 Mb	Cleft lip, omphalocele and possible ventricular septal defect		
33	arr[GRCh37] 10q22.2q26.3(75104407_135403394)x3,12p13.33(196821_3049321)x1	60.30 Mb 2.85 Mb	Multiple fetal anomalies, absent right eye, anhydramnios, fetal cardiac anomaly, and renal agenesis		Unconfirmed/possible unbalanced translocation
34	arr[GRCh37] 10q24.2q25.1(100390936_111580841)x1	11.19 Mb	Fetal double outlet right ventricle, possible abnormal cavum septum pellucidum, and possible club foot		
35	arr[GRCh37] 11q23.3q25(120009365_134928920)x1,12p13.33p11.1(190600_34078208)x3	14.92 Mb 33.91 Mb	Lemon‐shaped head, bilateral pyelectasis, suspected cardiac anomaly, and two‐vessel cord		Unconfirmed/possible unbalanced translocation
36	arr[GRCh37] 12p13.31(6050580_6237865)x1	187.28 kb	Miscarriage at 12 weeks		
37&38	arr(13)x3 [2]	95.53 Mb	(1) Recurrent Pregnancy Loss (2) Trisomy 13 suspicion		
39	arr[GRCh37] 13q22.1q34(74548020_115091801)x3	40.54 Mb	Two‐vessel cord and possible polydactyly		
40	arr[GRCh37] 14q11.2q24.3(20467750_74939762)x3	54.47 Mb	Bilateral renal agenesis and possible Dandy Walker malformation		
41	arr[GRCh37] 14q12q21.3(25186368_48277574)x1	23.09 Mb	Suspected poor fetal growth, transposition of great arteries, and absent cavum septum pellucidum		
42	arr[GRCh37] 15q11.2(22822019_23082298)x1	260.28 Kb	Ventricular septal defect	15q11.2 deletion syndrome	
43–46	arr[GRCh37] 15q11.2(22822019_23085218)x1 [4]	263.20 Kb	(1) Abnormal cell‐free fetal DNA screen, 1/17 risk trisomy 13/18, triploidy (2) Thick nuchal fold (3) Intrauterine fetal demise, omphalocele, cardiac defects, Dandy‐Walker malformation, hydrocephalus, bilateral ventriculomegaly, and dilation of third ventricle (4) Fetal brain abnormalities	15q11.2 deletion syndrome	
47	arr[GRCh37] 15q11.2(22822019_23085219)x1	263.20 Kb	Two‐vessel cord and diaphragmatic hernia	15q11.2 deletion syndrome	
48	arr[GRCh37] 15q11.2q13.3(22822019_32438944)x3,(18)x3	9.62 Mb 77.87 Mb	Missed abortion at 12 weeks	Trisomy 18	48,XY,+18,+mar
49&50	arr(16)x3 [2]	90.02 Mb	(1) Complete/unspecified spontaneous abortion without complication (2) Missed abortion	Trisomy 16	
51	arr[GRCh37] 16p13.11p11.2(16271313_31960103)x3	15.69 Mb	Abnormal ultra sound with concern for Down syndrome, hypoplastic 5th digit, absent nasal bone		
52	arr[GRCh37] 16p11.2(29657389_30192622)x1	535.23 Kb	Agenesis of kidney, multicystic dysplastic kidney, and anhydramnios	16p11.2 deletion syndrome	
53	arr[GRCh37] 16p11.2(29664618_30192346)x1	527.73 Kb	Intrauterine fetal demise	16p11.2 deletion syndrome	
54	arr[GRCh37] 16p11.2(28488583_29046251)x1	557.67 Kb	Intrauterine growth restriction and cardiac defect	16p11.2 deletion syndrome	
55	arr[GRCh37] 17p13.3(48858_2652026)x3,21q22.12q22.3(37539314_48091215)x1	2.60 Mb 10.55 Mb	Congenital heart defect, two‐vessel cord, and mild ventriculomegaly		der(21)t(17;21)(p13.3;q22.12)
56	arr[GRCh37] 17p12(14104475_15420103)x3	1.32 Mb	Intrauterine fetal demise and intrauterine growth retardation		
57–59	arr(18)x3 [3]	77.87 Mb	(1) Rule out Tri‐18 (2) Intrauterine fetal demise [2]	Trisomy 18	
60	arr[GRCh37] 18p11.32p11.21(146484_14117327)x1,18q22.1q23(63753505_78013620)x3	13.97 Mb 14.26 Mb	Abnormal cell‐free fetal DNA screen with unreportable result for chromosome 18 due to atypical pattern		46,XY,(der18)(qter–>q22.3::p11.2–>qter)dn
61	arr[GRCh37] 18p11.31(3055378_3554164)x1	498.79 Kb	Holoprosencephaly		
62	arr[GRCh37] 18q22.3q23(69040488_78013620)x1	8.97 Mb	Fetal cleft lip		
63	arr[GRCh37] 19q13.33(48315467_48398143)x1	82.68 Kb	Intrauterine fetal demise and recurrent pregnancy loss		
64	arr[GRCh37] 20p13(71023_2741908)x3,21q22.13q22.3(39264638_48091216)x1	2.67 Mb 8.83 Mb	Intrauterine fetal demise		t(20;21)(p13;q22.1)mat
65–71	arr(21)x3 [7]	32.61 Mb	(1) Intrauterine fetal demise [3] (2) Suspected Turner syndrome, (3) Atrioventricular canal defect, concern for Down syndrome, and growth restriction (4) Recurrent pregnancy loss (5) Recurrent spontaneous abortions‐ maternal rob(13;21) translocation carrier	Trisomy 21	
72–74	arr(22)x3 [3]	33.65 Mb	(1) Missed abortion (2) Recurrent pregnancy loss [2]	Trisomy 22	
75	arr[GRCh37] 22q11.21(18641409_21460594)x3	2.82 Mb	Missed abortion	22q11.2 microduplication syndrome	
76	arr[GRCh37] 22q11.21(18919528_21460594)x1	2.54 Mb	Congenital heart defect and 2 vessel cord	22q11.2 deletion syndrome	
77	arr[GRCh37] 22q11.21(18919528_21460595)x1	2.54 Mb	Ambiguous genitalia, cardiac defects, and micrognathia	22q11.2 deletion syndrome	
78	arr[GRCh37] 22q11.21q11.22(21079171_22423120)x1	1.34 Mb	Neural tube defect, ventricular septal defect, holoprosencephaly, and cleft lip and palate	Distal 22q11.2 deletion syndrome	
79	arr[GRCh37] 22q11.23(23751142_24991690)x3	1.24 Mb	Intrauterine fetal demise	22q11.2 microduplication syndrome	
80^a^	arr(X)x1 mos	154.93 Mb	Intrauterine fetal demise	Mosaic Turner Syndrome	
81–84	arr(X)x1 [4]	154.93 Mb	(1) Fetal demise (2) Intrauterine fetal demise and cystic hygroma (3) Multiple spontaneous abortions (4) Intrauterine fetal demise, increased risk of monosomy X on cell‐free fetal DNA screen, congenital heart disease, hydrops, neural tube defect, and short long bones	Turner Syndrome	
85&86	arr[GRCh37] Xp22.33 or Yp11.32(296520_618260 or 246520_568260)x1 [2]	321.74 Kb	(1) Severe polyhydramnios (2) Spontaneous pregnancy loss		
87	arr[GRCh37] Xq26.2(132696710_132839168)x1	142.46 Kb	Thick nuchal, diaphragmatic hernia, echogenic bowel, and absent nasal bone		
88	arr(14,20)x3	86.8 Mb & 62.68 Mb	Fetal demise, abnormal aneuploidy screen, and increased risk for Trisomy 18		
89	arr(15,21)x3	79.57 Mb & 32.61 Mb	Recurrent Pregnancy Loss		
90	arr(X)x2,(1‐22)x3		Spontaneous abortion of di/di twins		
91&92	arr(X,1‐22)x3 [2]		(1) Recurrent pregnancy loss (2) Fetal abnormalities and congenital anomalies		
93	arr(X)x2,(Y)x1,(1‐22)x3		Complete or unspecified spontaneous abortion with other complications		

*Note*: [#] indicates the number of cases with the same finding. Xp22.3/Yp11.32 losses were reported on the X chromosome ideogram, one loss was identified in a female fetus and the second loss was identified in a male fetus. This table contains the list of all 93 samples with pathogenic CNV findings annotated in Figure 3, it includes hg19 coordinates, the CNV segmental sizes, clinical indications, and the known associated OMIM syndrome.

^a^ Indicates a mosaic CNV finding.

In this study, the largest‐sized CNV finding was identified in a POC sample from a 25‐year‐old pregnant individual with recurrent, spontaneous first trimester pregnancy loss (Case 28). The sample had two de novo findings: a 37.87 Mb terminal loss of 8p23.3‐8p11.23 and a 106.54 Mb gain of 8p11.21‐8q24.3. This resulted in a partial monosomy of 8p and trisomy of 8q, and portions of 8p. These microarray findings can be suggestive of structural chromosomal abnormalities, as such, some of the cases in this cohort had follow‐up cytogenetic testing (Table 1). For example, case 31 had microarray findings of 9q34.3 loss and 16q23.3‐q24.3 gain, subsequent cytogenetic testing demonstrated a derivative chromosome 9 resulting from an unbalanced translocation between chromosome 9 and 1, with a deletion of chromosome material from 9q34 and duplication of material from 16q23.

## 4 DISCUSSION

CMA has the valuable utility of identifying clinically significant genomic aberrations in the setting of fetal anomaly or fetal loss (Vora et al., 2016). Beginning in 2016, we saw a volume increase which may be explained by the addition of improved diagnostic prenatal testing education, an oligonucleotide to SNP‐based array switch which identifies uniparental isodisomy (UPD), mosaicism, and consanguinity that would otherwise be undetected on oligonucleotide arrays. To gain a deep understanding of how CMA has been utilized in the obstetrics setting, this study summarized the CMA results from 842 prenatal and POC samples over a span of ten years in a hospital reference laboratory. Array results were categorized by the testing indications provided by the submitting clinicians. We characterized the diagnostic yields and clinically significant findings based on distinctive features, such as ultrasound findings, and abnormal prenatal screening.

Our cohort demonstrate a diagnostic yield of 11.05%. When stratified by microarray platform, the more updated platform, Agilent 180k CGH+SNP array, had a slightly higher diagnostic yield (11.25%) than the older NimbleGen 135k platform (8.7%). The higher probe density in the Agilent array, which provided higher resolution in detection, may contribute to the improvement in diagnostic yield. Historically, maternal serum screening was offered to pregnant individuals to estimate the risks for common trisomies and neural tube defects with an accuracy of 80%–90% (Ross & Elias, 1997). Recent advances which allow for the use of cell‐free DNA (cfDNA) in prenatal screening have increased the common trisomy accuracy to 99% (Rose et al., 2022). Regardless of the performance metrics (specificity and sensitivity) of a screening test, a diagnostic test is needed to confirm the screening result due to the risk of false positives and false negatives. In this study, we found that 56 cases (6.6%) had a clinical indication of abnormal prenatal screening; of this category, 29 cases were abnormal maternal serum screening, and 27 cases were abnormal prenatal cell‐free DNA screening. We observed that the number of prenatal cases with indications for noninvasive prenatal screening had increased throughout the years, from zero cases in 2011 to 22 cases in 2020. The increase in prenatal cell‐free DNA screening cases is not unexpected, as this prenatal screening test is now recommended for pregnant individuals regardless of their age‐associated risk or risk of chromosomal abnormality (Dungan et al., 2023; Gregg et al., 2016).

None of the abnormal maternal serum screening cases (*n* = 29) yielded a pathogenic finding, while 11.1% (*n* = 3/27) of cases with an abnormal prenatal cell‐free DNA screening yielded a clinically significant finding including: monosomy X (*n* = 1), 15q11.2 loss (*n* = 1), and 18p loss with 18q duplication (*n* = 1). Both the monosomy X and the atypical chromosome 18 finding were indicated on prenatal screening and confirmed by microarray. It is perhaps surprising that more trisomies 13, 18, and 21 were not confirmed by CMA, as the prenatal screenings have been shown to have high sensitivities in previous studies, yet there are no standard laboratory reporting standards and details about false negatives are lacking in literature which provides a false sense of safety (Rose et al., 2022). However, it is difficult to speculate the reasons due to the low number of abnormal prenatal cell‐free DNA screening samples in this study. Additionally, confined placental mosaicism, maternal mosaicism, maternal CNVs, maternal malignancy, vanishing twin syndrome, acceptable fetal fractions threshold differences by platform, low sequencing coverage, and CNV size are all factors that are known to contribute to discordant prenatal cell‐free DNA screening results(Grömminger et al., 2014; Hartwig et al., 2017; Mao et al., 2014; Samura & Okamoto, 2020; Wang et al., 2014, 2022; Wang, Meng, et al., 2015; Wang, Sahoo, et al., 2015). This emphasizes the importance of diagnostic confirmation of all abnormal prenatal cell‐free DNA screening results for making informed clinical decisions and for possible clinical intervention (Cherry et al., 2017; Lebo et al., 2015; Li et al., 2020). Currently, ACMG recommends prenatal cell‐free DNA screening over traditional methods for all singleton and twin pregnancies for fetal trisomies 13, 18, 21 and sex chromosome aneuploidies, while ACOG recommends prenatal genetic screening for all pregnant patients (Dungan et al., 2023; Gregg et al., 2016).

Abnormal fetal ultrasound findings had previously been reported in 3.68 out of 1000 pregnancies, and are often associated with genetic diseases or an isolated finding (Drukker et al., 2021). The common trisomies (chromosomes 13, 18, 21), and microdeletion syndromes (e.g., 22q11.2 microdeletion and Cri‐du‐chat) frequently have characteristic ultrasound findings in cardiac, neurological, and gastrointestinal systems. Invasive testing like prenatal CMA is the diagnostic test to identify these disorders since they are likely unidentified in common prenatal screening tests due to the lower prevalence in the general population (Conner et al., 2014). In this study, 80.7% (*n* = 422/523) of all prenatal cases had an abnormal US testing indication, of which 8.3% (*n* = 35/422) of those cases had pathogenic findings. These findings are consistent with other previous studies with diagnostic yields ranging from 6.5% to 9.6% (Brady et al., 2014; Costa et al., 2022; Patterson et al., 2021; Shaffer et al., 2012) (Figure 2a,b). POC arrays had a diagnostic yield of 16.3% (*n* = 52/319), 20 of these 52 cases were due to fetal demise and stillbirths, 13 cases were due to missed and spontaneous abortions as expected, while only 12 cases were due to recurrent pregnancy losses (RPL). RPL is known to affect two to five percent of all couples, two or more RPLs are indications for diagnostic genetic testing, we likely identify a lower proportion in our cohort because chromosomal analysis is the recommended genetic testing, not CMA (El Hachem et al., 2017; Practice Committee of the American Society for Reproductive Medicine, 2012). This highlights the utility of genetic testing for POCs, as genetic testing can provide a genetic etiology for the pregnancy loss, while also providing families with useful tools like preimplantation genetic testing (PGT) or invasive diagnostic prenatal testing for future pregnancies.

Limitations of this study include a prenatal array cohort consisting of predominantly cultured amniocyte samples. CVS samples were not included in this study, as only amniocyte samples were validated for prenatal testing in our clinical laboratory. While both CVS and amniocentesis demonstrate similar accuracies for genetic testing, it is possible that CVS would detect confined placental mosaicism, that would otherwise not be detected by amniocenteses. The clinically significant findings described herein are restricted to the limited number of patient samples included in this study, thus may not represent the true disease prevalence found in the general population. Clinical indications for CMA were provided on the sample requisition by the submitting clinician and entered into the reporting system by laboratory accessioners, thus offering only a limited view of the clinical picture.

VUS and CNV findings with no known disease association are not described in detail, as they are outside the scope of this study. It is possible that the interpretation of some VUS findings could be reclassified over time. Moreover, a pathogenic finding may not completely explain the clinical features of the fetus, and thus CMA does not mark the end of the diagnostic odyssey. Clinicians may choose to order additional genetic testing including next‐generation sequencing (NGS) panel testing and whole genome sequencing to reach a genetic diagnosis (Slavotinek et al., 2023).

In conclusion, this study provides summative data on the diagnostic yield and clinically significant CNV findings identified in CMA testing in prenatal and POC arrays. Our data details the proportions of study indications and demonstrates the differences in pathogenic findings when comparing prenatal samples and POC samples. Given the uptake of NGS testing in obstetric practices, future studies would be warranted to understand how NGS may resolve CMA‐negative cases in prenatal and POC testing (Mellis et al., 2022).

## AUTHOR CONTRIBUTIONS

Marco L. Leung designed the research. Joie O. Olayiwola and Marco L. Leung performed the analysis; Daniel Koboldt, Joie O. Olayiwola, and Mohammad Marhabaie generated figures; Amy Siemon, Cortlandt Myers, Danielle Mouhlas, George Kyle, Joie O. Olayiwola, Kathleen M. Schieffer, Theodora Matthews, and Taylor Porter analyzed microarray results. Catherine Cottrell, Hui Mei, Melanie Babcock, Jesse Hunter, Kathleen M. Schieffer, Mariam T. Mathew, Shalini Reshmi, Yassmine Akkari, Ying‐Chen Claire Hou and Marco L. Leung signed out clinical array reports. Joie O. Olayiwola and Marco L. Leung drafted the article. Catherine Cottrell, Cortlandt Myers, Hui Mei, Jesse Hunter, Kathleen M. Schieffer, Melanie Babcock, Ying‐Chen Claire Hou, and Taylor Porter edited the article.

## FUNDING INFORMATION

Not applicable.

## CONFLICT OF INTEREST STATEMENT

C.C, H.M, J.H, K.M.S, M.B, M.L.L, M.T.M, S.R, Y.A, and Y.C.H serve as clinical laboratory directors who perform genetics and genomics analyses on a fee‐for‐service basis.

## ETHICAL COMPLIANCE

This study was approved by the Institutional Review Board Committee at Nationwide Children's Hospital (NCH), Columbus, OH (STUDY00001490).

## PATIENT CONSENT STATEMENT

Waiver of individual consent was approved by Nationwide Children's Hospital IRB.

## PERMISSION TO REPRODUCE MATERIAL FROM OTHER SOURCES

Not applicable.

## CLINICAL TRIAL REGISTRATION

N/A.

## Supporting information


Supplemental Table 1.

Supplemental Table 2.

Supplementary Figure 1.

## Data Availability

Redacted data is available upon request. However, no identifiable information will be provided.
