# Supplementary material for: Clinically significant findings in a decade‐long retrospective study of prenatal chromosomal microarray testing
Source: Mol Genet Genomic Med. 2024 Jan 23;12(3):e2349. doi: 10.1002/mgg3.2349 (PMC10958178; doi:10.1002/mgg3.2349)
Supplement: Supplementary file 3 — Supplementary Figure 1. [file MGG3-12-e2349-s003.pdf]

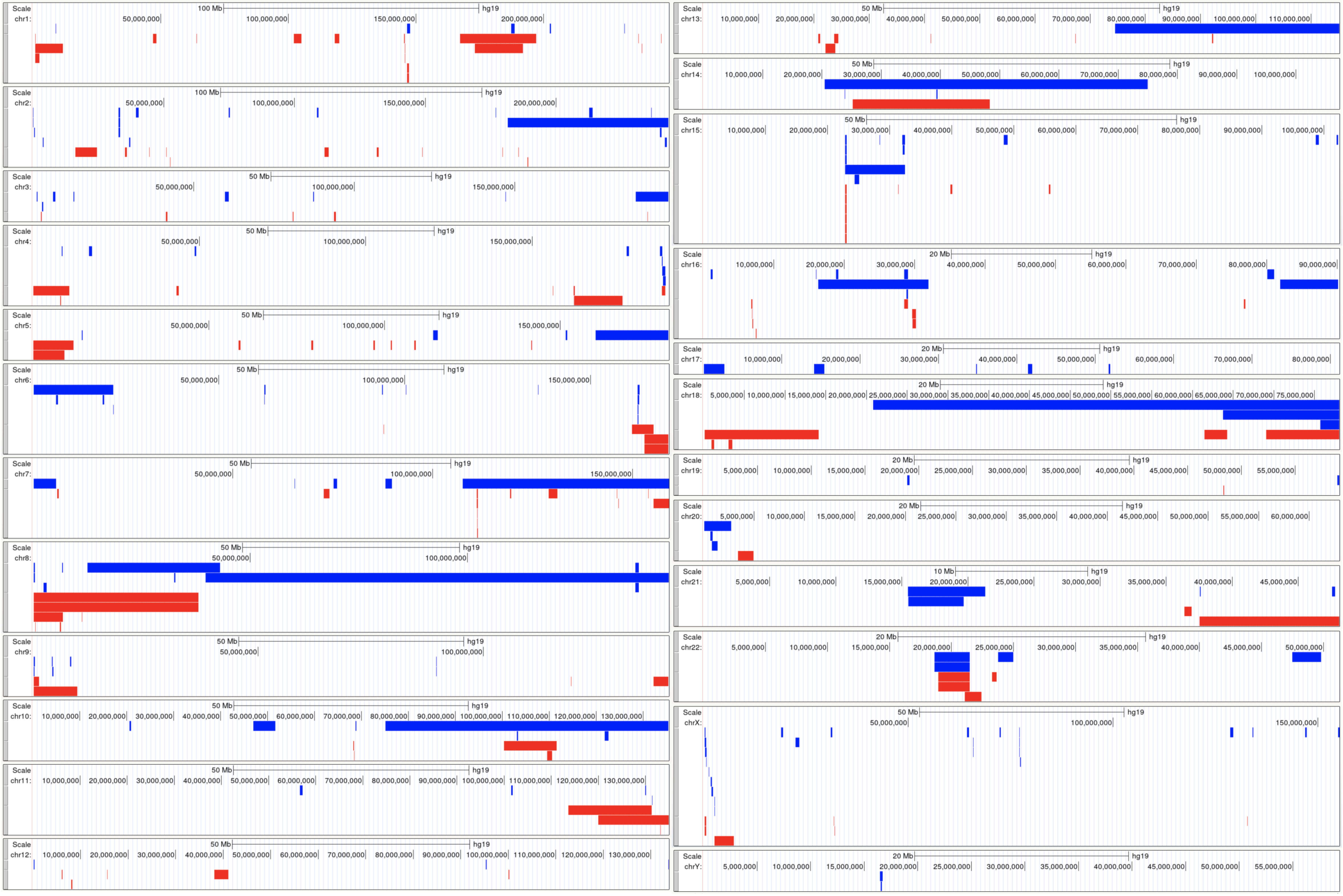

Supplementary Figure 1- Recurrent copy number losses and gains profile.

This profile represents all pathogenic and variants of unknown significance (VUS) copy number variants by chromosome number and hg19 genomic coordinates using the UCSC Genome Browser Tool which, excludes whole chromosome losses and gains. This figure is arranged by chromosome number (1-22) and the sex chromosomes are listed last. Each segment represents a CNV finding, but multiple segments in the same row correlate to different cases. Blue segments represent segmental gains and red segments represent segmental losses.
